# Supplementary material for: Genetically influenced tobacco and alcohol use behaviors impact erythroid trait variation
Source: medRxiv. 2023 May 11:2023.05.01.23289329. Preprint. [Version 2] doi: 10.1101/2023.05.01.23289329 (PMC10187333; doi:10.1101/2023.05.01.23289329)
Supplement: 1 [file NIHPP2023.05.01.23289329V2-supplement-1.pdf]

medRxiv preprint doi: https://doi.org/10.1101/2023.05.01.23289329; this version posted May 11, 2023. The copyright holder for this preprint (which was not certified by peer review) is the author/funder, who has granted medRxiv a license to display the preprint in perpetuity.
It is made available under a CC-BY 4.0 International license .

Supplementary Figures

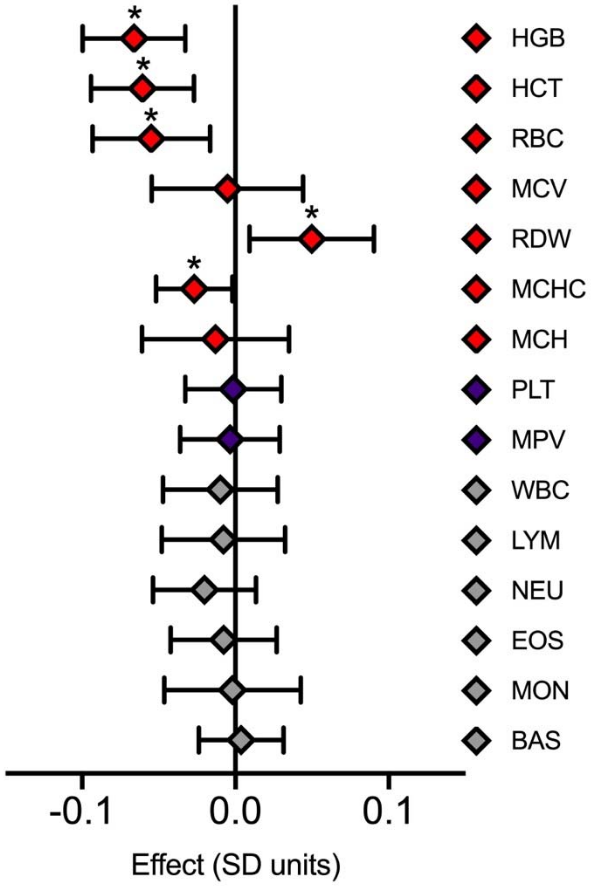

Supplementary Figure 1. Univariable MR effect estimates for genetically influenced SmkInit on the indicated blood traits. Effects of a 2-fold increase in SmkInit risk on the indicated blood traits by univariable MR. Bars indicate 95% confidence intervals. *p<0.05.

medRxiv preprint doi: https://doi.org/10.1101/2023.05.01.23289329; this version posted May 11, 2023. The copyright holder for this preprint (which was not certified by peer review) is the author/funder, who has granted medRxiv a license to display the preprint in perpetuity.
It is made available under a CC-BY 4.0 International license .

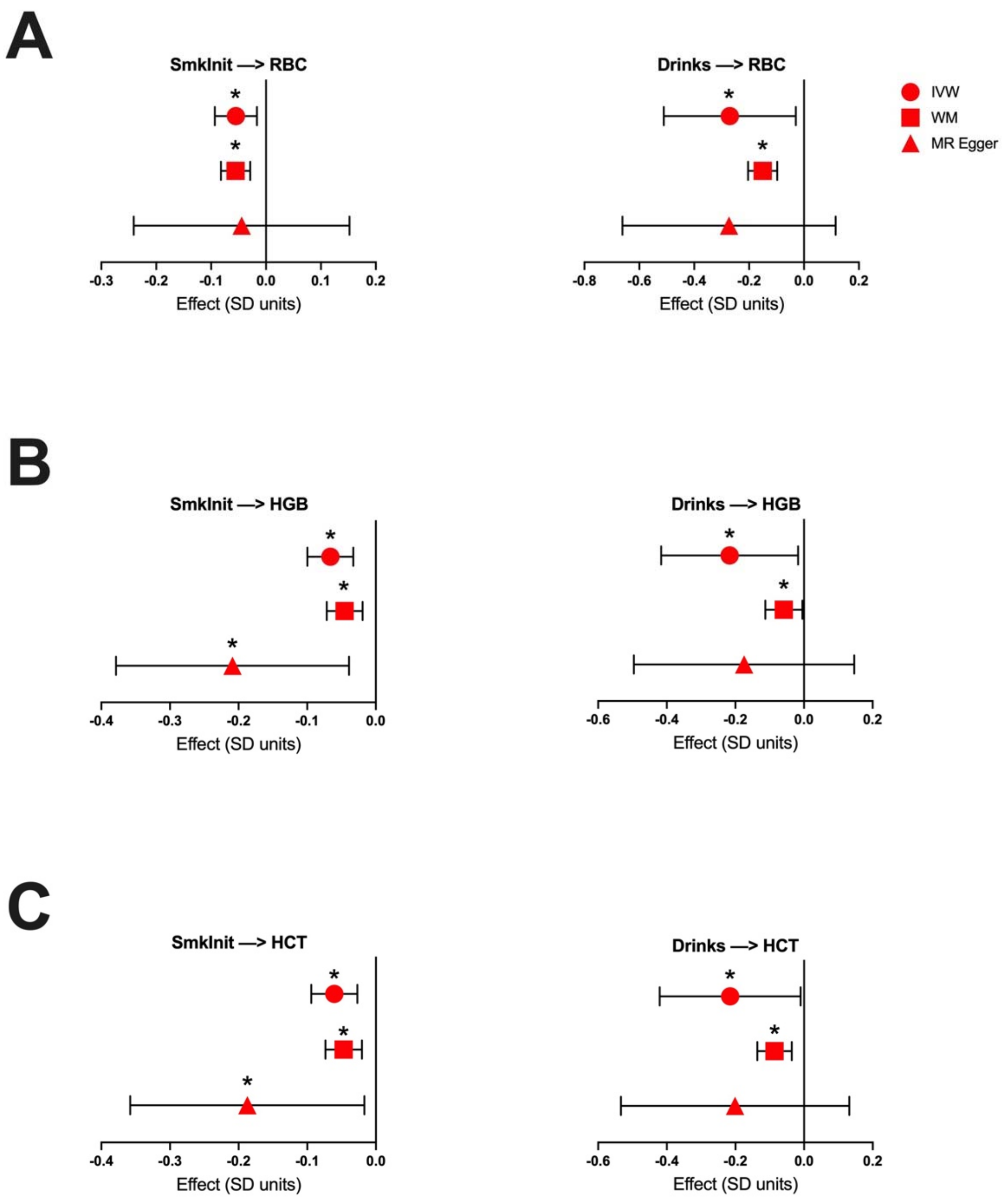

Supplementary Figure 2. Sensitivity analyses for trait association in univariable MR experiments.
(**A-C**) Effects of a 2-fold increase in SmkInit risk or a 1 SD unit increase in alcoholic drinks per week on (**A**) RBC, (**B**) HGB, or (**C**) HCT by weighted median (WM) and MR Egger regression analyses. IVW estimates also found in Figure 1 are presented here for comparison. Bars indicate 95% confidence intervals. *<0.05.

medRxiv preprint doi: https://doi.org/10.1101/2023.05.01.23289329; this version posted May 11, 2023. The copyright holder for this preprint (which was not certified by peer review) is the author/funder, who has granted medRxiv a license to display the preprint in perpetuity.
It is made available under a CC-BY 4.0 International license .

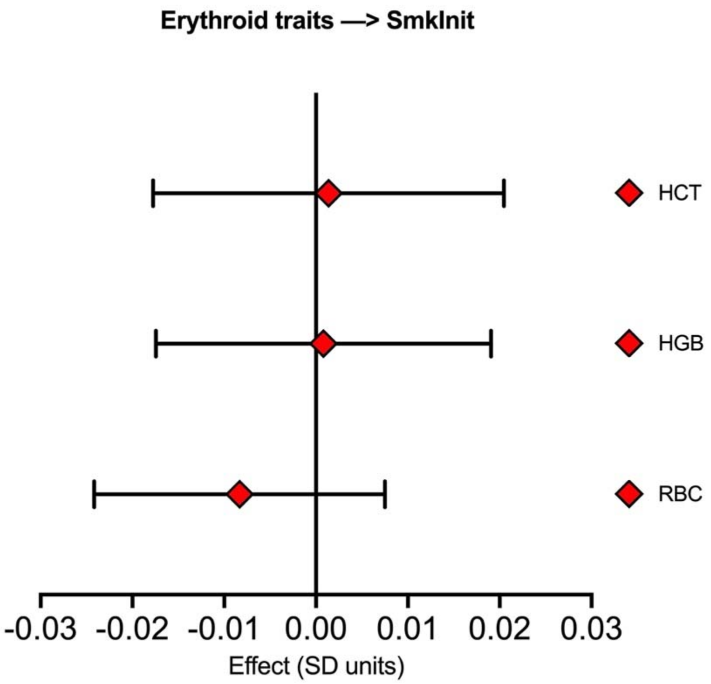

Supplementary Figure 3. Univariable MR effect estimates for a 1 SD unit increase in the indicated erythroid traits on SmkInit. None of the estimates reached statistical significance. Bars indicate 95% confidence intervals.

medRxiv preprint doi: https://doi.org/10.1101/2023.05.01.23289329; this version posted May 11, 2023. The copyright holder for this preprint (which was not certified by peer review) is the author/funder, who has granted medRxiv a license to display the preprint in perpetuity.
It is made available under a CC-BY 4.0 International license .

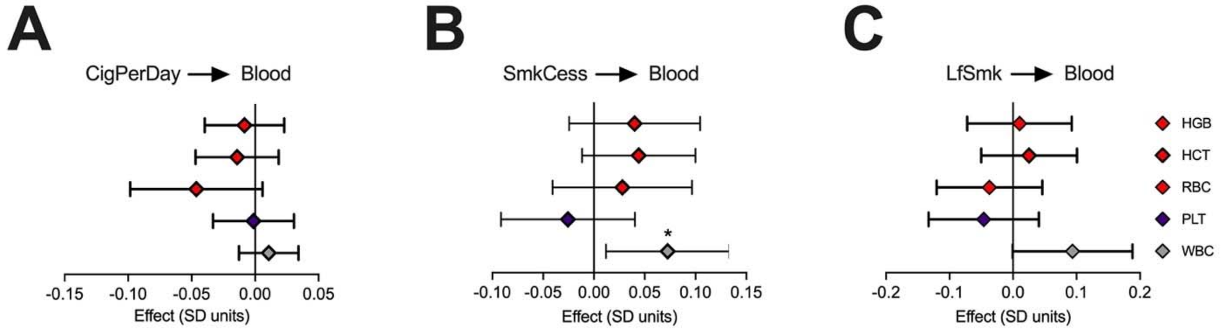

Supplementary Figure 4. Univariable MR effect estimates for a 1 SD unit increase in smoking traits on the indicated blood traits. Bars indicate 95% confidence intervals. *p<0.05.

medRxiv preprint doi: https://doi.org/10.1101/2023.05.01.23289329; this version posted May 11, 2023. The copyright holder for this preprint
(which was not certified by peer review) is the author/funder, who has granted medRxiv a license to display the preprint in perpetuity.
It is made available under a CC-BY 4.0 International license .

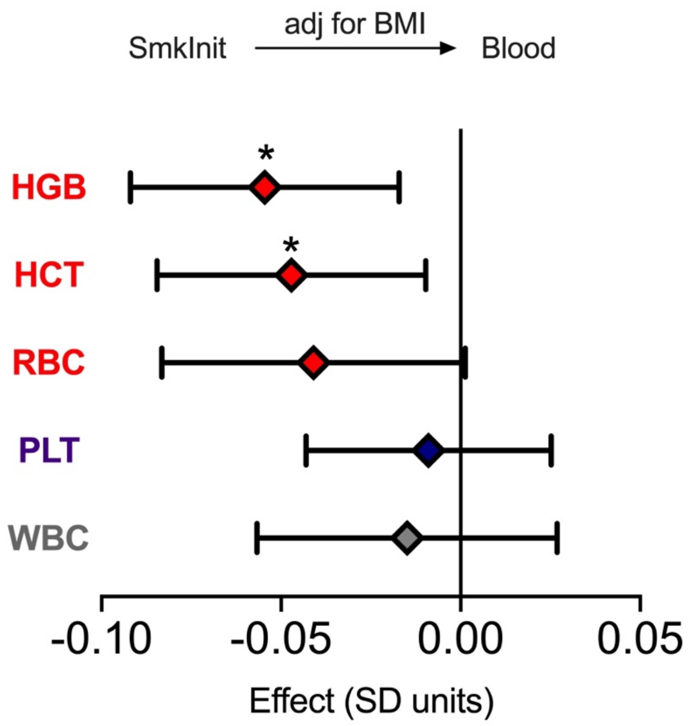

Supplementary Figure 5. MVMR experiments analyzing the effect of SmkInit on the indicated blood traits after adjusting for body mass index (BMI). Bars indicate 95% confidence intervals. *p<0.05.

medRxiv preprint doi: https://doi.org/10.1101/2023.05.01.23289329; this version posted May 11, 2023. The copyright holder for this preprint (which was not certified by peer review) is the author/funder, who has granted medRxiv a license to display the preprint in perpetuity.
It is made available under a CC-BY 4.0 International license .

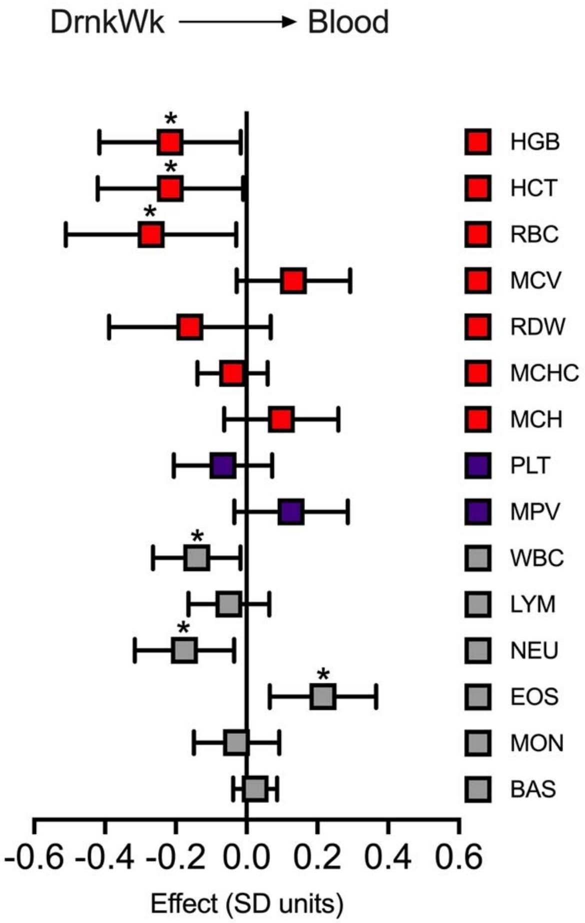

Supplementary Figure 6. Univariable MR effect estimates for a 1 SD unit increase in DrnkWk on the indicated blood traits. Bars indicate 95% confidence intervals. *p<0.05.

medRxiv preprint doi: https://doi.org/10.1101/2023.05.01.23289329; this version posted May 11, 2023. The copyright holder for this preprint (which was not certified by peer review) is the author/funder, who has granted medRxiv a license to display the preprint in perpetuity.
It is made available under a CC-BY 4.0 International license .

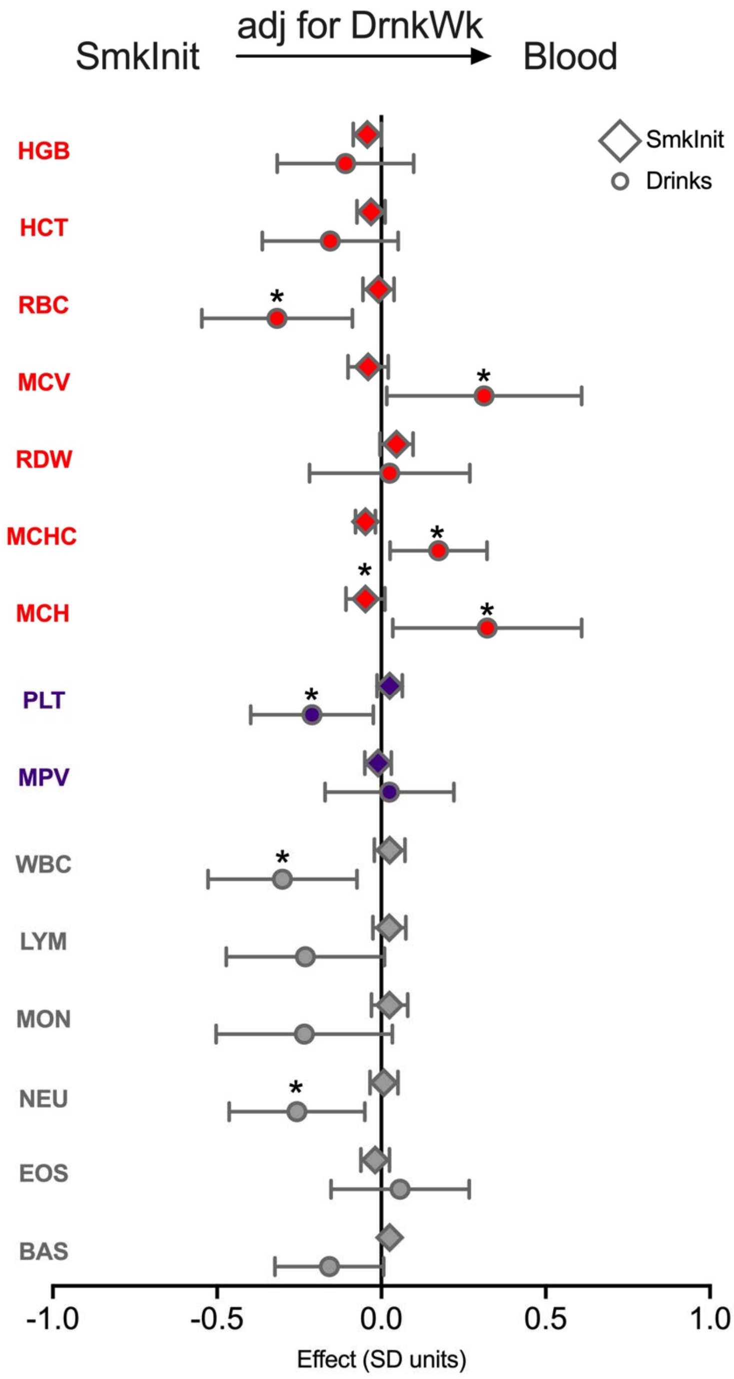

Supplementary Figure 7. MVMR effect estimates for SmkInit or DrnkWk on the indicated blood traits. All experiments used an instrumental variable for SmkInit adjusted for DrnkWk. After adjustment, SmkInit did not have significant effects on any blood trait whereas DrnkWk did retain some significant effects. Bars indicate 95% confidence intervals. *p<0.05.

medRxiv preprint doi: https://doi.org/10.1101/2023.05.01.23289329; this version posted May 11, 2023. The copyright holder for this preprint (which was not certified by peer review) is the author/funder, who has granted medRxiv a license to display the preprint in perpetuity.
It is made available under a CC-BY 4.0 International license .

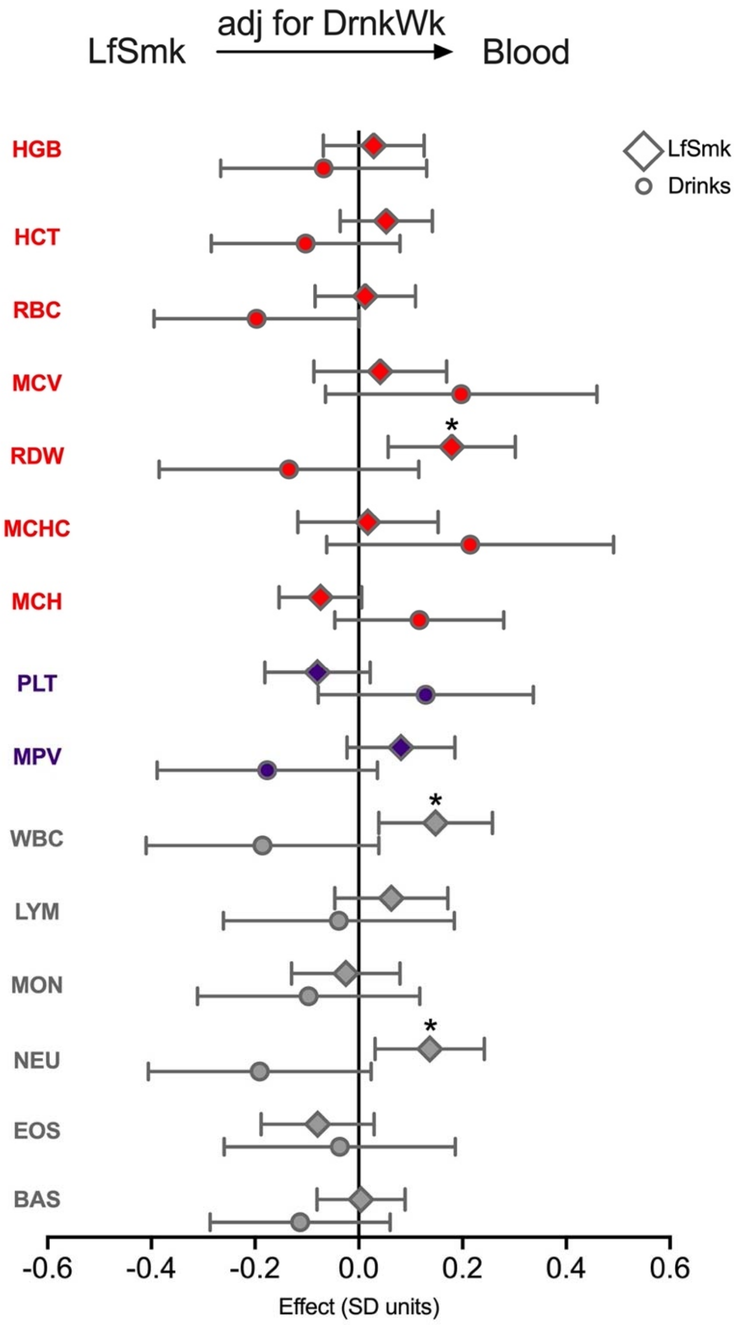

Supplementary Figure 8. MVMR effect estimates for LfSmk or DrnkWk on the indicated blood traits. All experiments used an instrumental variable for LfSmk adjusted for DrnkWk. After adjustment, LfSmk did not have significant effects on any blood trait whereas DrnkWk did retain some significant effects. Bars indicate 95% confidence intervals. *p<0.05.

medRxiv preprint doi: https://doi.org/10.1101/2023.05.01.23289329; this version posted May 11, 2023. The copyright holder for this preprint (which was not certified by peer review) is the author/funder, who has granted medRxiv a license to display the preprint in perpetuity.
It is made available under a CC-BY 4.0 International license .

Supplementary Tables

Supplementary Table 1. Explanations of genetically influenced traits analyzed in our study.

Supplementary Table 2. Craig-Donald F statistics for instrumental variables used in this study.
